# The relationship between increased air pollution expressed as PM_10_ concentration and the frequency of percutaneous coronary interventions in patients with acute coronary syndromes—a seasonal differences

**DOI:** 10.1007/s11356-020-08339-6

**Published:** 2020-04-08

**Authors:** Rafał Januszek, Bartłomiej Staszczak, Zbigniew Siudak, Jerzy Bartuś, Krzysztof Plens, Stanisław Bartuś, Dariusz Dudek

**Affiliations:** 1 Department of Clinical Rehabilitation, University of Physical Education, Krakow, Poland; 2 2nd Department of Cardiology and Cardiovascular Interventions, University Hospital, ul. Kopernika 17, 31-501, Krakow, Poland; 3 Jagiellonian University Medical College, Krakow, Poland; 4 Faculty of Medicine and Health Sciences, Jan Kochanowski University, Kielce, Poland; 5 KCRI, Krakow, Poland; 6 2nd Department of Cardiology, Jagiellonian University Medical College, Krakow, Poland; 7 Department of Interventional Cardiology, Jagiellonian University Medical College, Krakow, Poland

**Keywords:** Air pollution, Acute coronary syndromes, Percutaneous coronary interventions, Seasonal differences, Triggers

## Abstract

The aim of the presented study was to assess the relationship between air pollution expressed as particulate air matters less than 10 μm (PM_10_) and acute coronary syndromes (ACSs). In this observational study, we selected regions with low pollution according to PM_10_ (non-polluted) and with the highest pollution (polluted). The occurrence of percutaneous coronary interventions (PCIs) in patients with ACSs was matched according to the location. The current study included 7678 patients in polluted areas and 4327 patients from non-polluted regions. Analysing the period from January to December 2017, the number of patients undergoing angioplasty in monitored catheterization laboratories and the mean daily concentration of PM_10_ in all selected cities were calculated for each day. The annual average concentration of PM_10_ amounts to 50.95 μg/m^3^ in polluted and 26.62 μg/m^3^ in non-polluted cities (*P* < 0.01). The rise in PM_10_ pollution levels was related with the increased frequency of PCIs in patients with ACSs in polluted (*P* < 0.01) and non-polluted (*P* < 0.01) areas. In the non-polluted regions, the increase in PM_10_ concentration by every 1 μg/m^3^ causes 0.22 additional ACS angioplasties per week. In polluted regions, the same increase in PM_10_ concentration causes 0.18 additional ACS angioplasties per week. In non-winter weeks, the mean number of ACS PCIs expressed in promiles was lower than in winter weeks in polluted (*P* = 0.03) and non-polluted cities (*P* = 0.02). The study shows that the increase in air pollution expressed as PM_10_ concentration and winter time influences the frequency of ACS-related PCIs.

## Introduction

Comparative risk assessment of burden of disease and injury attributable to 67 risk factors and risk factor clusters in 21 regions of the world in 1990–2010 performed in the systematic analysis for the Global Burden of Disease Study 2010 demonstrated that in 2010, the three leading risk factors for global disease burden were high blood pressure, tobacco smoking including second-hand smoke, and alcohol use (Lim et al. 2012). There is also a fluctuation in risk factors in time, and so in 1990, among the three leading risk factors, there were being underweight in childhood, household air pollution from solid fuels and tobacco smoking, including second-hand smoke (Lim et al. 2012). The ambient particular matter pollution was assessed to be among the ten most influential risk factors ranked by attributable burden of disease (Lim et al. 2012). Analysis performed in 22 European cohorts within the multicentre European Study of Cohorts for Air Pollution Effects (ESCAPE) revealed that long-term exposure to fine particulate air pollution is associated with natural-cause mortality, also when considering concentration ranges below European annual mean upper limits (Beelen et al. 2014). It has been also demonstrated that deaths related to cardiovascular and respiratory system diseases, as well as cancers, remain in close relationship with air pollution (Beelen et al. 2014; Pothirat et al. 2019). Among the air pollutants, ambient particular matters play an important role, and so far, many studies have been completed assessing their impact on the incidence and pathogenesis of cardiovascular, respiratory and other deadly diseases that account for the largest percentage of deaths nowadays (Ye et al. 2001; Araujo et al. 2008; Madrigano et al. 2011; Nemmar et al. 2003). The influence of particular matter concentration on cardiac arrhythmias, hypertension, heart failure, stroke and coronary artery disease has been widely studied (Baccarelli et al. 2011; Breitner et al. 2019; Miller et al. 2007; Bai et al. 2019; Hong et al. 2002). The negative impact on oxidative stress, inflammatory processes and other mechanisms leading to the acceleration of the atherosclerotic process seem to be of key importance here (Sun et al. 2005; Bauer et al. 2010). It has been also shown that not only the degree of pollution with particular substances affects the above-mentioned coincidences but also other atmospheric parameters such as wind, humidity and air temperature, which may be a decisive factor triggering the occurrence of a given undesirable incident and related frequency of hospital admissions (Zanobetti and Peters 2015; Peng et al. 2008). A close relationship was found between the degree of air pollution and the frequency of acute coronary syndromes and hospitalizations, as well as cardiac arrest (Raza et al. 2014; Bhaskaran et al. 2009; Konduracka et al. 2019).

The aim of the current study was to assess the relationship between increased air pollution expressed as particulate air matters less than 10 μM (PM_10_) concentrations and the occurrence of acute coronary syndromes (ACSs) treated with percutaneous coronary interventions (PCIs). We also estimated the relationship between winter and non-winter periods and the frequency of ACSs treated with PCIs. The impact of other possible risk factors on the ACSs frequency treated with PCIs was also assessed.

## Methods

### Study population and data collection

**Fig. 1:**
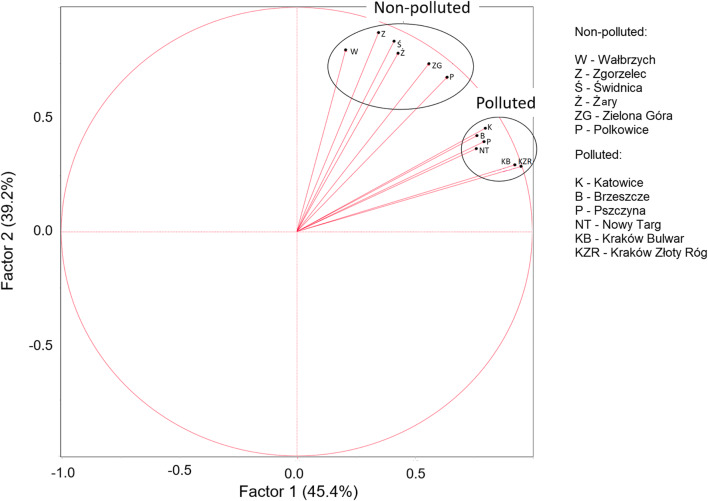
Biplot chart describing assignment of sites to non- and polluted regions to the extent of pollution expressed as PM_10_

**Fig. 2:**
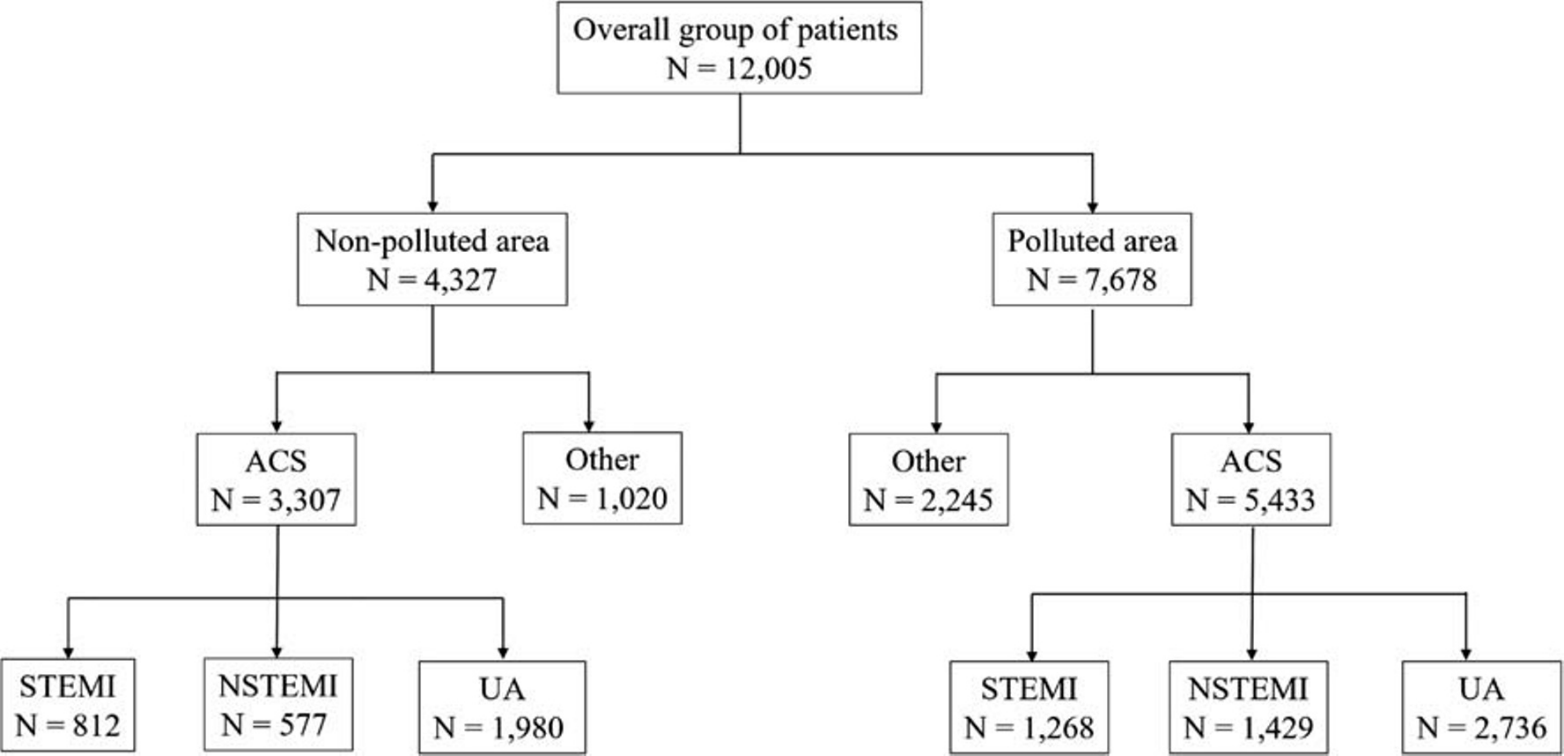
Patients flow-chart. NSTEMI non-ST segment elevation myocardial infarction, STEMI ST segment elevation myocardial infarction, UA unstable angina

**Fig. 3:**
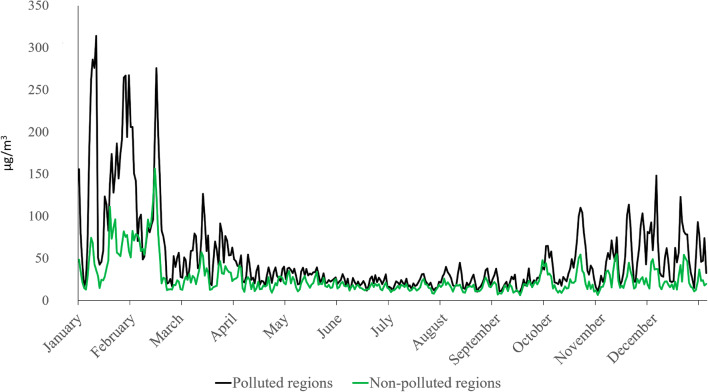
The relationship between air pollution and PM_10_ air concentration in the following months in 2017 in polluted and non-polluted regions

The study population was limited to the northern and north-western regions of Poland due to the fact that in this area, there are territories belonging to those with the largest air pollution and some with the best air quality considering the entire area of Poland and mean annual level of the most popular monitored pollutants. The purpose of this selection was to obtain the largest possible difference in average annual pollution values between two investigated groups of patients. Based on the data published by the Chief Inspectorate for Environmental Protection concerning the entire year 2017, we selected six cities (six catheter laboratories [cath labs]) with low pollution levels according to the average annual value of PM_10_ (‘non-polluted’) and five cities (six cath labs) with the highest pollution (‘polluted’). These locations served as a basis for the determination of 24/7 cath labs and the frequency of percutaneous coronary interventions in patients (PCIs) suffering from subsequent types of coronary artery disease, with special impact on the ACSs. Data on percutaneous coronary intervention practices in Poland were obtained from the ORPKI Polish National dataset. Although the ORPKI database is voluntary, the majority of all cath labs in Poland (98%) record their data in the registry. This was described in previously published manuscripts (Januszek et al. 2018a; b). Then, we matched the selected places to the location of the cath labs depending on the degree of pollution (Fig. 1). We have limited our calculations to people treated with PCIs due to ACSs. Patients’ clinical condition at admission to hospital and before PCI was assessed with the use of Killip-Kimball class (Killip 3rd and Kimball 1967). This classification consists of four grades: I class includes individuals with no clinical signs of heart failure; II class includes individuals with signs of pulmonary circulatory failure, which are expressed as rales or crackles in the lungs in auscultation, an S_3_ wave in electrocardiography and elevated jugular venous pressure visible as widening of jugular veins; III class describes individuals with acute pulmonary edema; and IV class describes individuals in cardiogenic shock or hypotension (measured as systolic blood pressure lower than 90 mmHg) and presence of peripheral vasoconstriction (oliguria, cyanosis, sweating). The patients’ flow charts are presented in Fig. 2. In the current nomenclature and for the purpose of the presented study, acute coronary syndromes consist of two types of acute myocardial infarctions (non-ST segment elevation myocardial infarction [NSTEMI] and ST segment elevation myocardial infarction [STEMI]) and unstable angina. Unstable angina refers to patients with acute and/or irregular chest pain and negative serum markers of myocardial necrosis, whereas STEMI usually refers to patients with acute total occlusion of epicardial coronary artery, and NSTEMI refers to other types of coronary artery stenoses or occlusions, both with significant elevation of serum markers of myocardial necrosis. Those definitions were made based on the actual guidelines of European Society of Cardiology (Thygesen et al. 2019). The register is accurate enough to match specific dates with the quality of observed air on the same day. The observed groups were also divided into winter and non-winter weeks due to the well-known fact that in winter weeks, the atmospheric air in selected regions is characterized by a significantly higher level of pollution at that time. This relation is presented in Fig. 3. The incidence of ACSs in the following days was determined on the basis of the mean ratio of urgent procedures to scheduled procedures in patients with stable angina. The current study counted 7678 patients in the polluted area and 4327 patients from the non-polluted regions treated with PCI and included patients with stable angina and ACSs. Analysing the period of 365 days, the number of patients undergoing angioplasty in monitored cath labs and the mean daily concentration of PM_10_ in all selected cities were calculated for each day. In order to better visualize air pollution trends and the frequency of performing PCI in patients with ACS, we have created new time intervals for weeks. Additionally, due to the difference in pollution levels, the analysed period was divided into winter (13 weeks—from December until March) and non-winter weeks (39 weeks—from March until December).

### Statistical analysis

The assignment of sites to non- and polluted areas was performed based on visual assessment of the biplot (Fig. 1) and mean PM_10_ concentration measures. Categorical variables are presented as numbers and percentages. Continuous variables are expressed as mean ± standard deviation (SD). Normality was assessed by the Shapiro-Wilk test. Equality of variances was assessed using Levene’s test. Differences between groups were compared using the Student’s or the Welch’s *t* test depending on the equality of variances for normally distributed variables. The Mann-Whitney U test was used for non-normally distributed continuous variables. Categorical variables were compared via Pearson’s chi-squared test or Fisher’s exact test if 20% of cells had an expected count less than 5. For two paired data samples, the paired Student’s *t* test was used if differences between pairs were normally distributed, and the Wilcoxon signed-rank was used if otherwise. To identify independent predictors of ACS subject status, univariate logistic regression analysis was performed on daily data with no lag provided in mean PM_10_ concentration. Associations between two variables were expressed as odds ratios (OR) along with 95% confidence intervals (95% CI). Linear regression models were used to check association between PM_10_ concentration and the number of procedures in sites for data aggregated per week. Normal distribution of residuals in the models was checked using the Shapiro-Wilk test. Heteroscedacity was checked using a median split for predicted values and examining whether the residuals in the upper half have different variability than those in the lower half using Levene’s test. Autocorrelation at lag 1 in the residuals was checked using the Durbin-Watson statistic. All *P* values were two-sided and considered statistically significant if below 0.05. All calculations were done with JMP®, Version 14.2.0 SAS Institute Inc., Cary, NC.

## Results

### Air pollution and frequency of ACSs treated with PCI

**Fig. 4:**
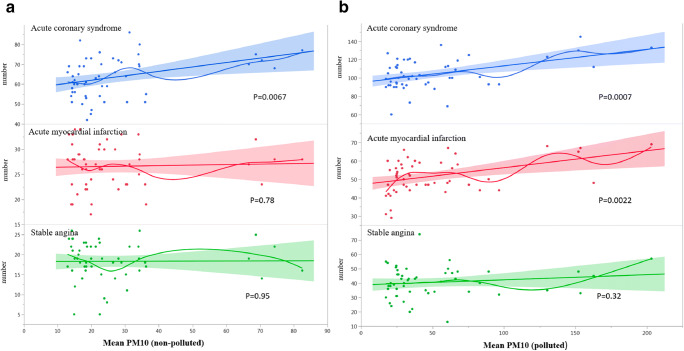
The relationship between PM_10_ air concentration and the frequency of stable anginas (SAs), acute myocardial infarctions (AMIs), and acute coronary syndromes (ACSs) in following weeks in non-polluted (**a**) and polluted (**b**) areas

The annual average concentration of PM_10_ amounts to 50.95 μg/m^3^ in polluted cities and 26.62 μg/m^3^ in non-polluted ones, which was significantly different (*P* < 0.01) (Figs. 1 and 3). It was proven that for both groups, the rise in PM_10_ pollution levels is connected with the increased frequency of PCIs in patients with ACSs (polluted *P* < 0.01 and non-polluted *P* < 0.01 areas). Moreover, we calculated that in the non-polluted regions, the increase in PM_10_ concentration by every 1 μg/m^3^ causes 0.22 (95% CI: 0.06–0.38, *P* = 0.007) additional ACS angioplasties per week. In polluted regions, the same increase in PM_10_ concentration causes 0.18 (95% CI: 0.08–0.28, *P* = 0.0007) additional ACS angioplasties per week (Fig. 4a, b).

### The frequency of ACSs treated with PCI and winter time

In non-winter weeks, the mean number of ACS PCIs expressed in promiles was lower than in winter weeks in polluted (18.56 ± 2.41 vs. 21.23 ± 3.98, *P* = 0.03) and non-polluted cities (18.68 ± 2.98 vs. 20.88 ± 2.57, *P* = 0.02).

### Patient characterization according to winter and non-winter weeks

Patients with ACSs treated with PCI during winter time tend to be burdened with higher rate of concomitant diseases, among which, there was a significantly higher rate of diabetics (*P* = 0.001) and smokers (*P* = 0.005). While the percentage of patients in Killip class I was significantly greater in patients treated during non-winter weeks (*P* = 0.0009), the percentage of patients in Killip class II was greater in patients treated during winter weeks (Table 1). Patients with ACSs treated with PCI during winter weeks presented higher incidences of multi-vessel disease and lower incidences of single-vessel disease in comparison to patients treated during non-winter-weeks. The blood flow before PCI assessed according to TIMI (Thrombolysis in Myocardial Infarction) grade was significantly less impaired in the group of patients treated during winter weeks as compared with non-winter weeks (*P* = 0.02). The mean radiation exposition was higher in patients treated during winter compared to non-winter weeks (*P* = 0.01; Table 2). The procedure-related complications tend to be higher in the group of patients treated during winter in comparison to non-winter weeks, but without statistical significance (*P* = 0.39). This was mainly sanctioned due to the higher rate of arterial dissections (*P* = 0.02; Table 3).

**Table 1:** General characteristics of patients with acute coronary syndromes treated with PCI according to winter and non-winter weeks in polluted and non-polluted regions

Selected indices	All patients, *N* = 8740	Winter weeks, *N* = 2408	Non-winter weeks, *N* = 6332	*P* value
Age (years)	67.3 ± 10.8	67.2 ± 10.7	67.3 ± 10.8	0.7
Gender, males (%)	68.1	68.5	68	0.65
Diabetes mellitus (%)	23.2	25.5	22.3	0.001
Arterial hypertension (%)	73.9	75.7	73.2	0.22
COPD (%)	2.54	2.61	2.51	0.78
Kidney failure (%)	6.45	6.97	6.25	0.21
Prior MI (%)	28.8	28.5	28.9	0.7
Cerebral stroke (%)	2.98	3.11	2.95	0.69
Prior PCI (%)	35.2	34	35.6	0.15
Prior CABG (%)	8.07	7.35	8.35	0.12
Smoking (%)	21.3	23.3	20.6	0.005
Killip class before PCI				
I	93.3	91.7	93.9	0.0009
II	3.97	5.65	3.31	< 0.0001
III	1.24	1.59	1.09	0.1
IV	1.49	1.15	1.63	0.14

*CABG* coronary artery bypass grafting, *COPD* chronic obstructive pulmonary disease, *MI* myocardial infarction, *PCI* percutaneous coronary intervention

**Table 2:** Procedure-related indices and coronary angiography of patients with ACS treated with PCI according to winter and non-winter weeks in polluted and non-polluted regions

	All patients, *N* = 8740	Winter weeks, *N* = 2408	Non-winter weeks, *N* = 6332	*P* value
Vascular access (%)				
Femoral	28.7	28.3	28.9	0.63
Radial left	13.8	13.1	14	0.27
Radial right	57.1	58	56.8	0.33
Other	0.4	0.6	0.3	0.07
Coronary angiography (%)				
Single vessel disease	52.4	44.8	48.2	0.009
MVD	41	47.9	44	0.002
MVD + LMCA	6.3	6.9	7.6	0.28
Isolated LMCA	0.3	0.4	0.2	0.27
TIMI flow before PCI (%)				
0	24.8	23.2	25.5	0.02
I	6.5	6.1	6.6	
II	15.8	15.8	15.8	
III	52.9	54.9	52.1	
TIMI flow after PCI (%)				
0	2.5	2.4	2.5	0.52
I	0.8	0.8	0.8	
II	3	3.3	2.9	
III	93.7	93.5	93.8	
IVUS, %	1.87	1.32	2.08	0.02
FFR, %	1.55	1.28	1.65	0.21
Thrombectomy, %	3.6	3.61	3.6	0.97
Radiation, Gy	0.991 ± 0.833	1.028 ± 0.828	0.977 ± 0.834	0.01
Contrast dose, mL	177.3 ± 82.1	177 ± 77.5	177.4 ± 83.8	0.81

*IVUS* intravascular ultrasound, *FFR* fractional-flow reserve, *LMCA* left main coronary artery, *MVD* multi-vessel disease, *PCI* percutaneous coronary intervention, *TIMI* thrombolysis in myocardial infarction

**Table 3:** Procedural related complications of patients with acute coronary syndrome treated with percutaneous coronary intervention according to winter and non-winter weeks in polluted and non-polluted regions

Type of procedural related complication	All patients, *N* = 8740	Winter weeks, *N* = 2408	Non-winter weeks, *N* = 6332	*P* value
Overall (%)	2.391	2.616	2.3	0.39
Myocardial infarction (%)	0.114	0.124	0.11	0.86
CAP (%)	0.205	0.166	0.221	0.61
Allergic reactions (%)	0.034	0.041	0.031	0.82
Bleedings (%)	0.274	0.041	0.363	0.01
No-reflow (%)	0.652	0.872	0.568	0.11
Cardiac arrest (%)	0.835	0.955	0.789	0.44
Death (%)	0.434	0.498	0.41	0.57
Dissection (%)	0.197	0.383	0.126	0.02
Cerebral stroke (%)	0.013	0.047	0	0.1

*CAP* coronary artery perforation

### Triggers of the occurrence of ACSs treated with PCIs

Among possible triggers of increased frequency of ACSs treated with PCI related to the greater air pollution expressed as PM_10_ air concentration, we estimated all indices and present and compare them in Tables 1, 2 and 3. Among factors significantly related to the rate of ACSs treated with PCI in the case of increased air pollution assessed by PM_10_ air concentration, we noticed non-winter weeks (OR: 0.959; 95% CI: 0.938–0.982, *P* = 0.0004), male gender (OR: 0.987; 95% CI: 0.977–0.997, *P* = 0.01), female gender (OR:1.026; 95% CI: 1.007–1.046, *P* = 0.006) and smoking (OR: 0.969; 95% CI: 0.952–0.986, *P* = 0.0006), while a borderline relationship was observed for prior coronary artery bypass grafting operations (OR: 1.032; 95% CI: 0.997–1.067, *P* = 0.06) and prior PCI (OR: 0.989; 95% CI: 0.977–1.001, *P* = 0.08). We also observed a trend for the rate of patients treated with PCI due to ACS with increased air pollution in patients with multi-vessel disease and left-main coronary artery involvement as well as in patients with chronic obstructive pulmonary disease, kidney failure or prior cerebral stroke (Table 4).

**Table 4:** The impact of selected indices on the frequency of acute coronary syndromes depending on the air pollution assessed as PM_10_

Selected indices	Odds ratio	95% Confidence interval	*P* value
Non-winter weeks	0.959	0.938–0.982	0.0004
Males	0.987	0.977–0.997	0.01
Females	1.026	1.007–1.046	0.006
Prior cerebral stroke	1.012	0.974–1.051	0.539
Diabetes mellitus	0.987	0.971–1.004	0.14
Prior CABG	1.032	0.997–1.067	0.06
Prior PCI	0.989	0.977–1.001	0.08
Prior myocardial infarction	0.992	0.979–1.007	0.27
Smoking	0.969	0.952–0.986	0.0006
Hypertension	0.995	0.986–1.005	0.41
COPD	1.018	0.968–1.07	0.47
Multi-vessel disease	1.011	0.992–1.029	0.23
LMCA involvement	4.878	0.617–38.528	0.13
Multi-vessel disease and LMCA	1.02	0.972–1.07	0.41
Kidney failure	1.013	0.983–1.044	0.38

*CABG* coronary artery bypass grafting, *COPD* chronic obstructive pulmonary disease, *LMCA* left main coronary artery, *PCI* percutaneous coronary intervention

## Discussion

The main finding of the current study is that increased air pollution, independent of location (polluted or not-polluted regions), is related to the increased rate of PCIs in patients with ACS. Moreover, we also demonstrated that the same increase in air pollution expressed as PM_10_ is related to a higher increase in the rate of ACSs treated with PCI in non-polluted compared with the polluted regions. The third finding of the presented study is that the frequency of ACSs treated with PCI in patients with ACSs was higher in winter than non-winter weeks in polluted and non-polluted areas. The current analysis also revealed that among factors increasing the negative impact of air pollution on the rate of ACSs treated with PCI were female gender, prior PCI and coronary artery bypass grafting surgery, while among factors that decreased that effect were smoking and non-winter weeks. There was also a trend for the increase of ACSs treated with PCI parallel with increased air pollution for patients with multi-vessel coronary artery disease, left main-coronary artery involvement, prior PCI, chronic obstructive pulmonary disease and kidney failure.

Triggers of acute coronary infarctions have been examined in several studies, and among factors associated with the onset of acute myocardial infarction, intense exercise or physical exertion, diet, coffee and alcohol consumption, sexual activity, cocaine or marijuana abuse, emotional stress and environmental conditions have been distinguished (Nawrot et al. 2011). Environmental conditions most often included temperature, influenza epidemics and air pollution (Wang et al. 2019). Also, other cardiovascular diseases accompanying coronary artery disease, such as heart failure or arrhythmias, were found to predispose admissions in urgent mode due to exacerbation of ischemic heart disease (Mittleman et al. 1993). So far, physical effort and emotional stress were considered to be the main triggers of acute coronary syndromes (Mann et al. 2002).

Previously published papers regarding the relationship between temperature and the presence of acute coronary syndromes have shown a higher frequency of their occurrence as the temperature decreases (Wichmann et al. 2012; Danet et al. 1999; Bhaskaran et al. 2010). These studies concerned regions with potentially higher air pollution in winter seasons, in which the air temperature was lower. However, in one study performed in a relatively less polluted area from Finland, it was shown that the temperature relationship with the incidence of acute coronary syndromes is lower for regions with a lower average annual temperature than for regions with an average annual temperature and with greater temperature drops during winter periods, including northern European continental countries (Barnett et al. 2005). However, studies conducted in areas with a much warmer year-round climate, such as Los Angeles, have shown seasonal dependence. And in the case of the autumn and winter period, a higher incidence of admission to hospitals was noted due to cardiopulmonary diseases, and this was explained by atmospheric stagnation (Linn et al. 2000).

So far, a number of studies have been published regarding both short- and long-term exposure to air pollution and their impact on acute cardiovascular events (Kim et al. 2019). It has been shown in a large meta-analysis performed by Tan et al. that legislative changes contributing to the reduction of long-term exposure to smoke have impact on reducing the incidence of acute cardiovascular and respiratory events (Tan and Glantz 2012). Also, studies conducted among large areas in the USA have shown that reducing exposure to ambient fine matter of air pollution contributed to improvement in life expectancy (Pope 3rd et al. 2009). In the current study, we evaluated the short-term effects of PM_10_ pollution on the frequency of ACSs treated with PCI. In a Boston study and other studies, a relationship was found between the short-term increase in air pollution (hours) and the increased incidence of ACS rate (Peters et al. 2001, Pan et al. 2019 and Chen et al. 2020). The relationship of the extent of air pollution expressed as PM_10_ concentration has also been demonstrated in other countries with a warmer climate (Cendon et al. 2006). In regions such as São Paulo, the relationship between the daily temperature and the incidence of acute myocardial infarctions was also shown, and this relationship was linear, U-shaped with the lowest incidence for the temperature of 22 °C (Sharovsky et al. 2004). A similar linear relationship between daily temperature and the incidence of adverse events was demonstrated in other parts of the world, such as the Netherlands or Taiwan (Kunst et al. 1993; Pan et al. 1995).

The relationship of lower temperatures with the increased incidence of ACSs, resulting from the increased emission of pollutants associated with the heating season, seems to have decisive impact. But other factors should also be taken into account, such as arterial vasoconstrictor response to cold air or the increased incidence of infections in seasons with lower air temperatures. On the other hand, the increased temperature may contribute, for example, to dehydration of patients, which leads to the emergence of clinically silent stenoses in the coronary arteries and subsequent symptomatic acute coronary syndromes, to which the elderly seem particularly vulnerable. The relationship between age and the rate of ACSs treated with PCI after adjustment to increased air pollution has not been confirmed in the current analysis. However, the study performed in Tokyo did not demonstrate a relationship between cardiovascular transports to hospital and increased temperature, except for the increased rate of pneumonias and decreased number of transports due to hypertension (Ye et al. 2001). On the contrary, a study performed in Denver demonstrated that higher temperatures were related to an increase in the frequency of hospitalization for acute myocardial infarction and congestive heart failure. However, at the same time, this study suggested that higher temperatures are associated with a decrease in the frequency of visits due to coronary atherosclerosis and pulmonary heart disease and have no significant effect on admissions because of cardiac dysrhythmias (Koken et al. 2003).

Among other factors related to the incidence of acute coronary syndromes, in addition to temperature and air pollution, such a relationship has been demonstrated for a number of factors, including age, gender, concomitant diseases including chronic respiratory diseases, infections and the time of day and week (Ye et al. 2001; Sharovsky et al. 2004; Pan et al. 1995; Koken et al. 2003; Barnett et al. 2006; Smeeth et al. 2004). One of the studies performed in USA, which included > 300,000 patients hospitalized due to MI, confirmed that older people, those with atrial fibrillation, COPD, diabetes, heart failure, pneumonia and males, were more prone on increased air pollution expressed as PM_10_. In this analysis, the authors used time-stratified controls matched according to day of the week or temperature to detect possible residual confounding by weather (Zanobetti and Schwartz 2005). Also, the European Society of Cardiology, in its consensus entitled ‘Expert Position Paper on Air Pollution and Cardiovascular Diseases’, included a sub-section on the interaction between air pollution and traditional risk factors (Newby et al. 2015). It has been underlined that, for example, obese patients and those with diabetes are at increased risk of effects of air pollution with PM2.5 (Brook et al. 2009). This was mainly attributed to impaired insulin sensitivity by air pollutants (Rajagopalan and Brook 2012). A similar relationship was demonstrated for hypertensive patients (Brook et al. 2009; Szyszkowicz et al. 2012). Also, complications mediated by air pollutions associated with hypertension in pregnant women seem to be more frequent (van den Hooven et al. 2011). Those relationships were not confirmed in our analysis. To the contrary, we noticed that male gender and smoking were a protecting factor in patients with increased exposition to air pollution expressed as PM_10_ concentration. This remains in line with other calculations, which demonstrate that patients living in polluted regions were less prone to the effects of increased air pollution in terms of increased level of ACSs treated with PCI. However, not confirmed by logistic regression analysis, we noticed that the trend that patients with more advanced and disseminated coronary artery lesions with left main coronary artery involvement and more often patent target coronary artery are more prone to increased air pollution and at increased risk of ACSs treated with PCI, but without statistical significance. This also remains in close relationship with higher rate of procedural related complications and radiation exposure, mainly due to increased rate of coronary artery dissections, which is typical for endovascular treatment of more advanced and disseminated atherosclerotic lesions, especially with the involvement of the left main coronary artery.

## Conclusions

The annual average PM_10_ concentration was significantly higher in polluted cities compared with unpolluted cities. In both polluted and unpolluted areas, a rise in PM_10_ concentration was significantly associated with a greater frequency of PCI. Patients in cities with clean air were more sensitive to pollution rises, with each 1 μg/m^3^ increase in PM_10_ concentration linked to 0.22 additional PCIs per week. While in polluted cities, the same rise in PM_10_ was linked with just 0.18 additional PCIs per week. Regarding the seasonal effect, the PCI rate was significantly lower in non-winter, compared with winter, weeks in both polluted and clean cities.

## References

[CR1] Araujo JA, Barajas B, Kleinman M, Wang X, Bennett BJ, Gong KW, Navab M, Harkema J, Sioutas C, Lusis AJ, Nel AE (2008). Ambient particulate pollutants in the ultrafine range promote early atherosclerosis and systemic oxidative stress. Circ Res.

[CR2] Baccarelli A, Barretta F, Dou C, Zhang X, McCracken JP, Díaz A, Bertazzi PA, Schwartz J, Wang S, Hou L (2011). Effects of particulate air pollution on blood pressure in a highly exposed population in Beijing, China: a repeated-measure study. Environ Health.

[CR3] Bai L, Shin S, Burnett RT, Kwong JC, Hystad P, van Donkelaar A, Goldberg MS, Lavigne E, Copes R, Martin RV, Kopp A, Chen H (2019). Exposure to ambient air pollution and the incidence of congestive heart failure and acute myocardial infarction: a population-based study of 5.1 million Canadian adults living in Ontario. Environ Int.

[CR4] Barnett AG, Dobson AJ, Mcelduff P, Salomaa V, Kuulasmaa K, Sans S, WHO MONICA Project (2005). Cold periods and coronary events: an analysis of populations worldwide. J Epidemiol Community Health.

[CR5] Barnett AG, Williams GM, Schwartz J, Best TL, Neller AH, Petroeschevsky AL, Simpson RW (2006). The effects of air pollution on hospitalizations for cardiovascular disease in elderly people in Australian and New Zealand cities. Environ Health Perspect.

[CR6] Bauer M, Moebus S, Möhlenkamp S, Dragano N, Nonnemacher M, Fuchsluger M, Kessler C, Jakobes H, Memmesheimer M, Erbel R, Jöckel KH, Hoffmann B, HNR Study Investigative Group (2010). Urban particulate matter air pollution is associated with subclinical atherosclerosis: results from the HNR (Heinz Nixdorf Recall) study. J Am Coll Cardiol.

[CR7] Beelen R, Raaschou-Nielsen O, Stafoggia M, Andersen ZJ, Weinmayr G, Hoffmann B, Wolf K, Samoli E, Fischer P, Nieuwenhuijsen M, Vineis P, Xun WW, Katsouyanni K, Dimakopoulou K, Oudin A, Forsberg B, Modig L, Havulinna AS, Lanki T, Turunen A, Oftedal B, Nystad W, Nafstad P, De Faire U, Pedersen NL, Östenson CG, Fratiglioni L, Penell J, Korek M, Pershagen G, Eriksen KT, Overvad K, Ellermann T, Eeftens M, Peeters PH, Meliefste K, Wang M, Bueno-de-Mesquita B, Sugiri D, Krämer U, Heinrich J, de Hoogh K, Key T, Peters A, Hampel R, Concin H, Nagel G, Ineichen A, Schaffner E, Probst-Hensch N, Künzli N, Schindler C, Schikowski T, Adam M, Phuleria H, Vilier A, Clavel-Chapelon F, Declercq C, Grioni S, Krogh V, Tsai MY, Ricceri F, Sacerdote C, Galassi C, Migliore E, Ranzi A, Cesaroni G, Badaloni C, Forastiere F, Tamayo I, Amiano P, Dorronsoro M, Katsoulis M, Trichopoulou A, Brunekreef B, Hoek G (2014). Effects of long-term exposure to air pollution on natural-cause mortality: an analysis of 22 European cohorts within the multicentre ESCAPE project. Lancet.

[CR8] Bhaskaran K, Hajat S, Haines A, Herrett E, Wilkinson P, Smeeth L (2009). Effects of air pollution on the incidence of myocardial infarction. Heart.

[CR9] Bhaskaran K, Hajat S, Haines A, Herrett E, Wilkinson P, Smeeth L (2010). Short term effects of temperature on risk of myocardial infarction in England and Wales: time series regression analysis of the Myocardial Ischaemia National Audit Project (MINAP) registry. BMJ.

[CR10] Breitner S, Peters A, Zareba W, Hampel R, Oakes D, Wiltshire J, Frampton MW, Hopke PK, Cyrys J, Utell MJ, Kane C, Schneider A, Rich DQ (2019). Ambient and controlled exposures to particulate air pollution and acute changes in heart rate variability and repolarization. Sci Rep.

[CR11] Brook RD, Rajagopalan S, Pope CA, Brook JR, Bhatnagar A, Diez-Roux AV, Holguin F, Hong Y, Luepker RV, Mittleman MA, Peters A, Siscovick D, Smith SC, Whitsel L, Kaufman JD, American Heart Association Council on Epidemiology and Prevention, Council on the Kidney in Cardiovascular Disease, and Council on Nutrition, Physical Activity and Metabolism (2009). Particulate matter air pollution and cardiovascular disease: An update to the scientific statement from the American Heart Association. Circulation.

[CR12] Brook RD, Urch B, Dvonch JT, Bard RL, Speck M, Keeler G, Morishita M, Marsik FJ, Kamal AS, Kaciroti N, Harkema J, Corey P, Silverman F, Gold DR, Wellenius G, Mittleman MA, Rajagopalan S, Brook JR (2009). Insights into the mechanisms and mediators of the effects of air pollution exposure on blood pressure and vascular function in healthy humans. Hypertension.

[CR13] Cendon S, Pereira LA, Braga AL, Conceição GM, Cury Junior A, Romaldini H, Lopes AC, Saldiva PH (2006). Air pollution effects on myocardial infarction. Rev Saude Publica.

[CR14] Chen K, Schneider A, Cyrys J, Wolf K, Meisinger C, Heier M, von Scheidt W, Kuch B, Pitz M, Peters A, Breitner S, KORA Study Group (2020). Hourly exposure to ultrafine particle metrics and the onset of myocardial infarction in Augsburg, Germany. Environ Health Perspect.

[CR15] Danet S, Richard F, Montaye M, Beauchant S, Lemaire B, Graux C, Cottel D, Marécaux N, Amouyel P (1999). Unhealthy effects of atmospheric temperature and pressure on the occurrence of myocardial infarction and coronary deaths. A 10-year survey: the Lille-World Health Organization MONICA project (monitoring trends and determinants in cardiovascular disease). Circulation.

[CR16] Pothirat C, Chaiwong W, Liwsrisakun C, Bumroongkit C, Deesomchok A, Theerakittikul T, Limsukon A, Tajarernmuang P, Phetsuk N (2019). Acute effects of air pollutants on daily mortality and hospitalizations due to cardiovascular and respiratory diseases. J Thorac Dis.

[CR17] Hong YC, Lee JT, Kim H, Ha EH, Schwartz J, Christiani DC (2002). Effects of air pollutants on acute stroke mortality. Environ Health Perspect.

[CR18] Januszek R, Siudak Z, Dziewierz A, Rakowski T, Dudek D, Bartuś S (2018). Chronic obstructive pulmonary disease affects the angiographic presentation and outcomes of patients with coronary artery disease treated with percutaneous coronary interventions. Pol arch intern med.

[CR19] Januszek R, Dziewierz A, Siudak Z, Rakowski T, Dudek D, Bartuś S (2018). Chronic obstructive pulmonary disease and periprocedural complications in patients undergoing percutaneous coronary interventions. PLoS One.

[CR20] Killip T, Kimball JT (1967). Treatment of myocardial infarction in a coronary careunit. A two year experience with 250 patients. Am J Cardiol.

[CR21] Kim IS, Yang PS, Lee J, Yu HT, Kim TH, Uhm JS, Kim JY, Pak HN, Lee MH, Joung B (2019). Long-term fine particulate matter exposure and cardiovascular mortality in the general population: a nationwide cohort study. J Cardiol pii.

[CR22] Koken PJ, Piver WT, Ye F, Elixhauser A, Olsen LM, Portier CJ (2003). Temperature, air pollution, and hospitalization for cardiovascular diseases among elderly people in Denver. Environ Health Perspect.

[CR23] Konduracka E, Niewiara Ł, Guzik B, Kotynia M, Szolc P, Gajos G, Nessler J, Podolec P, Żmudka K (2019). Effect of short-term fluctuations in outdoor air pollution on the number of hospital admissions due to acute myocardial infarction among inhabitants of Kraków, Poland. Pol Arch Intern Med.

[CR24] Kunst AE, Looman CW, Mackenbach JP (1993). Outdoor air temperature and mortality in the Netherlands: a time-series analysis. Am J Epidemiol.

[CR25] Lim SS, Vos T, Flaxman AD, Danaei G, Shibuya K, Adair-Rohani H, Amann M, Anderson HR, Andrews KG, Aryee M, Atkinson C, Bacchus LJ, Bahalim AN, Balakrishnan K, Balmes J, Barker-Collo S, Baxter A, Bell ML, Blore JD, Blyth F, Bonner C, Borges G, Bourne R, Boussinesq M, Brauer M, Brooks P, Bruce NG, Brunekreef B, Bryan-Hancock C, Bucello C, Buchbinder R, Bull F, Burnett RT, Byers TE, Calabria B, Carapetis J, Carnahan E, Chafe Z, Charlson F, Chen H, Chen JS, Cheng AT, Child JC, Cohen A, Colson KE, Cowie BC, Darby S, Darling S, Davis A, Degenhardt L, Dentener F, Des Jarlais DC, Devries K, Dherani M, Ding EL, Dorsey ER, Driscoll T, Edmond K, Ali SE, Engell RE, Erwin PJ, Fahimi S, Falder G, Farzadfar F, Ferrari A, Finucane MM, Flaxman S, Fowkes FG, Freedman G, Freeman MK, Gakidou E, Ghosh S, Giovannucci E, Gmel G, Graham K, Grainger R, Grant B, Gunnell D, Gutierrez HR, Hall W, Hoek HW, Hogan A, Hosgood HD, Hoy D, Hu H, Hubbell BJ, Hutchings SJ, Ibeanusi SE, Jacklyn GL, Jasrasaria R, Jonas JB, Kan H, Kanis JA, Kassebaum N, Kawakami N, Khang YH, Khatibzadeh S, Khoo JP, Kok C, Laden F, Lalloo R, Lan Q, Lathlean T, Leasher JL, Leigh J, Li Y, Lin JK, Lipshultz SE, London S, Lozano R, Lu Y, Mak J, Malekzadeh R, Mallinger L, Marcenes W, March L, Marks R, Martin R, McGale P, McGrath J, Mehta S, Mensah GA, Merriman TR, Micha R, Michaud C, Mishra V, Mohd Hanafiah K, Mokdad AA, Morawska L, Mozaffarian D, Murphy T, Naghavi M, Neal B, Nelson PK, Nolla JM, Norman R, Olives C, Omer SB, Orchard J, Osborne R, Ostro B, Page A, Pandey KD, Parry CD, Passmore E, Patra J, Pearce N, Pelizzari PM, Petzold M, Phillips MR, Pope D, Pope CA, Powles J, Rao M, Razavi H, Rehfuess EA, Rehm JT, Ritz B, Rivara FP, Roberts T, Robinson C, Rodriguez-Portales JA, Romieu I, Room R, Rosenfeld LC, Roy A, Rushton L, Salomon JA, Sampson U, Sanchez-Riera L, Sanman E, Sapkota A, Seedat S, Shi P, Shield K, Shivakoti R, Singh GM, Sleet DA, Smith E, Smith KR, Stapelberg NJ, Steenland K, Stöckl H, Stovner LJ, Straif K, Straney L, Thurston GD, Tran JH, Van Dingenen R, van Donkelaar A, Veerman JL, Vijayakumar L, Weintraub R, Weissman MM, White RA, Whiteford H, Wiersma ST, Wilkinson JD, Williams HC, Williams W, Wilson N, Woolf AD, Yip P, Zielinski JM, Lopez AD, Murray CJ, Ezzati M, MA AM, Memish ZA (2012). A comparative risk assessment of burden of disease and injury attributable to 67 risk factors and risk factor clusters in 21 regions, 1990–2010: a systematic analysis for the Global Burden of Disease Study 2010. Lancet.

[CR26] Linn WS, Szlachcic Y, Gong H, Kinney PL, Berhane KT (2000). Air pollution and daily hospital admissions in metropolitan Los Angeles. Environ Health Perspect.

[CR27] Madrigano J, Baccarelli A, Mittleman MA, Wright RO, Sparrow D, Vokonas PS, Tarantini L, Schwartz J (2011). Prolonged exposure to particulate pollution, genes associated with glutathione pathways, and DNA methylation in a cohort of older men. Environ Health Perspect.

[CR28] Mann JK, Tager IB, Lurmann F, Segal M, Quesenberry CP, Lugg MM, Shan J, De Eeden V (2002). Air pollution and hospital admissions for ischemic heart disease in persons with congestive heart failure or arrhythmia. Environ Health Perspect.

[CR29] Miller KA, Siscovick DS, Sheppard L, Shepherd K, Sullivan JH, Anderson GL, Kaufman JD (2007). Long-term exposure to air pollution and incidence of cardiovascular events in women. N Engl J Med.

[CR30] Mittleman MA, Maclure M, Tofler GH, Sherwood JB, Goldberg RJ, Muller JE (1993). Triggering of acute myocardial infarction by heavy physical exertion. Protection against triggering by regular exertion. Determinants of myocardial infarction onset study investigators. N Engl J Med.

[CR31] Nawrot TS, Perez L, Künzli N, Munters E, Nemery B (2011). Public health importance of triggers of myocardial infarction: a comparative risk assessment. Lancet.

[CR32] Nemmar A, Hoet PH, Dinsdale D, Vermylen J, Hoylaerts MF, Nemery B (2003). Diesel exhaust particles in lung acutely enhance experimental peripheral thrombosis. Circulation.

[CR33] Newby DE, Mannucci PM, Tell GS, Baccarelli AA, Brook RD, Donaldson K, Forastiere F, Franchini M, Franco OH, Graham I, Hoek G, Hoffmann B, Hoylaerts MF, Künzli N, Mills N, Pekkanen J, Peters A, Piepoli MF, Rajagopalan S, Storey RF, ESC Working Group on Thrombosis, European Association for Cardiovascular Prevention and Rehabilitation; ESC Heart Failure Association (2015). Expert position paper on air pollution and cardiovascular disease. Eur Heart.

[CR34] Pan HY, Cheung SM, Chen FC, Wu KH, Cheng SY, Chuang PC, Cheng FJ (2019). Short-term effects of ambient air pollution on ST-elevation myocardial infarction events: are there potentially susceptible groups?. Int J Environ Res Public Health.

[CR35] Pan WH, Li LA, Tsai MJ (1995). Temperature extremes and mortality from coronary heart disease and cerebral infarction in elderly Chinese. Lancet.

[CR36] Peng RD, Chang HH, Bell ML, McDermott A, Zeger SL, Samet JM, Dominici F (2008). Coarse particulate matter air pollution and hospital admissions for cardiovascular and respiratory diseases among Medicare patients. JAMA.

[CR37] Peters A, Dockery DW, Muller JE, Mittleman MA (2001). Increased particulate air pollution and the triggering of myocardial infarction. Circulation..

[CR38] Pope CA, Ezzati M, Dockery DW (2009). Fine-particulate air pollution and life expectancy in the United States. N Engl J Med.

[CR39] Rajagopalan S, Brook RD (2012). Air pollution and type 2 diabetes: mechanistic insights. Diabetes..

[CR40] Raza A, Bellander T, Bero-Bedada G, Dahlquist M, Hollenberg J, Jonsson M, Lind T, Rosenqvist M, Svensson L, Ljungman PL (2014). Short-term effects of air pollution on out-of-hospital cardiac arrest in Stockholm. Eur Heart J.

[CR41] Sharovsky R, César LA, Ramires JA (2004). Temperature, air pollution, and mortality from myocardial infarction in São Paulo, Brazil. Braz J Med Biol Res.

[CR42] Smeeth L, Thomas SL, Hall AJ, Hubbard R, Farrington P, Vallance P (2004). Risk of myocardial infarction and stroke after acute infection or vaccination. N Engl J Med.

[CR43] Sun Q, Wang A, Jin X, Natanzon A, Duquaine D, Brook RD, Aguinaldo JG, Fayad ZA, Fuster V, Lippmann M, Chen LC, Rajagopalan S (2005). Long-term air pollution exposure and acceleration of atherosclerosis and vascular inflammation in an animal model. JAMA.

[CR44] Szyszkowicz M, Rowe BH, Brook RD (2012). Even low levels of ambient air pollutants are associated with increased emergency department visits for hypertension. Can J Cardiol.

[CR45] Tan CE, Glantz SA (2012). Association between smoke-free legislation and hospitalizations for cardiac, cerebrovascular, and respiratory diseases: a meta-analysis. Circulation.

[CR46] Thygesen K, Alpert JS, Jaffe AS, Chaitman BR, Bax JJ, Morrow DA, White HD, ESC Scientific Document Group (2019). Fourth universal definition of myocardial infarction (2018). Eur Heart J.

[CR47] van den Hooven EH, de Kluizenaar Y, Pierik FH, Hofman A, van Ratingen SW, Zandveld PY, Mackenbach JP, Steegers EA, Miedema HM, Jaddoe VW (2011). Air pollution, blood pressure, and the risk of hypertensive complications during pregnancy: the generation R study. Hypertension.

[CR48] Wang M, Hopke PK, Masiol M, Thurston SW, Cameron S, Ling F, van Wijngaarden E, Croft D, Thevenet-Morrison K, Chalupa D, Rich DQ (2019). Changes in triggering of ST-elevation myocardial infarction by particulate air pollution in Monroe County. New York over time: a case-crossover study Environ Health.

[CR49] Wichmann J, Ketzel M, Ellermann T, Loft S (2012). Apparent temperature and acute myocardial infarction hospital admissions in Copenhagen, Denmark: a case-crossover study. Environ Health.

[CR50] Ye F, Piver WT, Ando M, Portier CJ (2001). Effects of temperature and air pollutants on cardiovascular and respiratory diseases for males and females older than 65 years of age in Tokyo, July and august 1980-1995. Environ Health Perspect.

[CR51] Zanobetti A, Peters A (2015). Disentangling interactions between atmospheric pollution and weather. J Epidemiol Community Health.

[CR52] Zanobetti A, Schwartz J (2005). The effect of particulate air pollution on emergency admissions for myocardial infarction: a multicity case-crossover analysis. Environ Health Perspect.

